# Dance Movement Therapy with Children: Practical Aspects of Remote Group Work

**DOI:** 10.3390/children9060870

**Published:** 2022-06-11

**Authors:** Einat Shuper-Engelhard, Maya Vulcan

**Affiliations:** 1 Graduate School of Creative Art Therapies, Faculty of Social Welfare and Health Sciences, Emili Sagol Creative Arts Therapies Research Center, University of Haifa, Haifa 31905, Israel; 2 Head, Dance Movement Therapy Program, Graduate School of Creative Art Therapies, University of Haifa, Haifa 31905, Israel; 3 Graduate School of Creative Art Therapies, Faculty of Humanities and Social Sciences, Kibbutzim College of Education, Tel Aviv 62507, Israel; mayavulcan@gmail.com; 4 Head, Dance Movement Therapy Program, Graduate School of Creative Art Therapies, Faculty of Humanities and Social Sciences, Kibbutzim College of Education, Technology and the Arts, Tel Aviv 62507, Israel; 5 Graduate School of Creative Art Therapies, Faculty of Social Welfare and Health Sciences, University of Haifa, Haifa 31905, Israel

**Keywords:** COVID-19, dance movement therapy, remote therapy

## Abstract

The global COVID-19 outbreak has forced psychotherapists to find creative ways to continue treating their clients from afar. Dance movement therapy emphasizes the body–mind connection and offers a unique mode of emotional intervention for supporting mental processes. The present study is the first to examine the distinctive qualities of group dance movement therapy in the context of remote emotional intervention with young children. Fourteen preschool children participated in six DMT meetings. The data generated three themes: 1. play as a platform for transforming technical complexity into an expression of the inner world; 2. accessories and props as means of motivation for movement and imaginative play; 3. playfulness-inhibiting conditions in settings of remote therapy. The discussion examines the significance of bodily expressions in remote therapy for understanding the needs of children in times of crisis and for getting acquainted with feelings and sensations which do not lend themselves easily to verbal expression.

## 1. Group Dance Movement Therapy with Children

Group dance movement therapy (DMT) aims at enabling a sensory and creative experience, using movement, play, and the arts [1]. Each session addresses individual goals, alongside the enhancement of group awareness, interaction, and cohesion [2], and introduces healing elements such as expression, rhythm, synchrony, vitalization, integration, cohesion, and symbolism [3]. Group DMT with children involves free movement, dance, and play, which allow for the discharge of psychomotor energy, symbolization, and projection [4]. Dance movement therapists use various methods, such as creative and expressive dance and movement, role-playing, gross and perceptual motor activities, and a blend of improvised and structured movement experiences [2].

Research has indicated that DMT groups provide experience in creating, organizing, and processing meaningful effective and cognitive information, and are especially significant for elementary-school-aged children [5], inasmuch as most developmental tasks occur in a group context [6]. Belonging to a group helps the child’s process of separation from his/her parents and offers a safe and supportive space for developing relationships and identification with one’s peer group. Furthermore, a sense of belonging affords the participants an experience of resilience and is an important step in self-development.

Group DMT with children has been found to be applicable with various conditions and in diverse situations, for example: children diagnosed with ASD [7,8], emotionally disturbed children [9], young children and adolescents in a psychiatric unit [2,10], youngsters with learning difficulties [1,11], children suffering from earthquake trauma [4], proactive work with adolescents [12], withdrawn adolescents [13], and refugee children [14].

The group therapist needs to consider developmental processes related to abilities of self-control of psychomotor impulses, expression of emotions, self-adaptation to external stimuli (music and use of space), and the expression of empathy at a young age [6]. A preliminary study of children suffering from earthquake trauma in Taiwan found that group DMT for children in elementary school requires an active presence on the part of the therapists during the session, and they should aim at creating a potential space for playing, imagining, and processing the meanings inherent in symbolization and expression [4]. The session includes references to movements, feelings, emotions, and spontaneous emotional experiences, along with structured guided activities and the use of projective props (balls, scarves, ropes, pillows, etc.) that encourage movement and verbal mediation, which accompanies the in-session movement. The use of props supports creative expression, enriches the spectrum of associations, and offers sensory stimulation which augments the qualities of movement. The use of props can also contribute to the creation of new and more complex experiences of the self, and to the expression of group processes [4,10]. Working through DMT with children under the age of five and their families, Lykou [1] stresses the importance of accompanying the children with an unconditionally positive attitude, and to allow them to move as they please without expecting a specific “correct” move. Moreover, the therapeutic work should take place mainly through movement and play rather than through verbal discourse and discussion of insights, as these capacities are still limited at the age of latency.

Group DMT with children is often based on the work and structure offered by Marian Chace (1896–1970) and is comprised of various stages: The initial phase is established through physical warm-up, from which the central theme of the session derives and develops in the second stage of exploration. The third and final stage of each session is devoted to gathering and sharing the participants’ experience and is characterized by a decrease in the level of psychophysical activity and a verbal discussion of the movement experience [2]. The basic assumption of this model is that the consistent, standardized, and predictable structure of the sessions provides a sense of security and trust in the therapist, which allows the participants to engage in movement and personal exploration processes [2,5].

## 2. Remote Psychotherapy with Children

The global COVID-19 pandemic has induced therapists to adjust their work settings in accordance with the pandemic restrictions and to transfer the therapeutic work from physical settings to virtual remote digital platforms. These adjustments have highlighted the challenges involved in remote psychotherapy for children, in view of their limited attention span and greater need of mobility and non-verbal communication [15].

Fonagy, Campbell, Truscott, and Fuggle (2020) emphasize that in “face-to face” work the therapist responds to both explicit and implicit communications with the child and makes sense of these communications by creating mental models of the intentional state of the client in a remarkably fluid way. When working remotely, however, the adoption of the mentalizing stance of not knowing is even more critical, as the therapist has less access to those implicit forms of communication [16].

The literature on the particularities of remote group psychotherapy for children addresses four obstacles faced by the therapist: First, the treatment framework now depends on the client’s access to proper and private space. Second, the virtual environment hinders existing unconscious regulatory processes which occur at times of physical presence, as both bodies impact each other in physical space. Third, the therapeutic presence of the therapists for their clients is obviously qualitatively different from that of a conventional setting. The fourth obstacle relates to the elements that enter the screen and do not belong to the group-therapeutic scenario. These factors are usually ignored, whereas in interpersonal therapy they would be given a dynamic interpretation [5].

At the same time, in remote psychotherapy for children, the home environment provides therapists with an opportunity to get to know the child’s reality outside the clinic and watch interactions with siblings and parents at home as they really occur and in real time [15]. Through the acquaintance with the child’s favorite objects, an empathetic view of his/her physical actions (such as rolling, jumping, and dancing), a view of objects produced by the child, and a discussion of the meanings he/she attributes to them, a new intimacy is created between the child and his/her therapist (ibid.). These advantages notwithstanding, the physical absence of the therapist entails major disadvantages: the difficulty in maintaining a therapeutic alliance, the lack of eye contact and the actual, concrete gaze of the therapist at his/her client, the discomfort of the therapists themselves, and difficulties in expressing and conveying empathy, warmth, and sensitivity from afar [17].

These insights, albeit grounded in general clinical experience and theoretical understanding, have yet to be more fully validated through specific research on early childhood. A study involving 28 children and adolescents found that remote CBT is just as, and sometimes even more effective, than face-to-face CBT therapy in reducing symptoms of depression [18]. Additionally, numerous studies describe achievements in remote psychotherapy of children and adolescents with depression [19] and anxiety [20] and various other conditions [21].

In the field of creative arts therapies, Potash et al. (2020) [22] suggest that creative arts therapists can support public health psychosocial guidelines by disseminating information, promoting expression and inspiration, challenging stigma, securing family connections, monitoring secondary traumatic stress, developing coping strategies and resilience, maintaining relationships, and contributing to the enhancement of a sense of hope. Spooner’s (2019) [23] insights concerning remote creative arts therapy highlight the advantages of this mode of therapy as a way to strengthen the connections with family and community.

Recent research findings emphasize the importance of creative arts therapies in the education system, both in day-to-day routine and in times of crisis. Despite the numerous difficulties, the study highlighted the therapists’ ability to maintain significant contact with their young clients and their parents, and with the educational staff. The factors that support the success of the therapy are defining a therapeutic contract and managing setting and process, as well as creative thinking and support from colleagues and supervisors [24]. These findings reinforce insights which emerge from case studies in the field [20,21,22,23] and reviews of therapists’ experience around the world [25].

Dance movement therapy is based on the body and its visible movement, but this inherent and essential quality is severely challenged when working remotely and is even more complicated when working with young children. There are few studies which focus on the field of remote DMT. An article which describes DMT by phone with psychiatric clients emphasizes the difficulty in creating synchronized experiences and the continuous need to choose between seeing the client’s facial expressions or his/her full body movement [26]. Case studies show that in order to continue sustaining DMT during COVID-19, the therapist must observe all the clients’ actions as a dynamic expression of the mind, including their response to computer disengagement, disappearance from the screen, exposure or hiding of certain body parts, etc. [27]. The choice of body parts revealed on the screen, for example, signifies the ability to organize oneself within a new reality, to take into account the other, and convey experiences related to body image and the perception of body boundaries.

During remote psychotherapy sessions clients are often preoccupied with questions of whether their therapist can see, hear, feel, guard, and protect them—perhaps even more powerfully than in the physical therapy room, which often serves as a guarded space in and of itself [27].The dance movement therapist may suggest that the client move his or her body within the confines of the screen and explore possibilities in front of the camera and the room, and each client may respond to the suggestion in his or her own way. An offer to get closer or farther away from the screen will bring with it countless new stories and new ways of communication. Reference to the physicality of the room (proximity to the wall, space or lack thereof, bed, soft/rigid objects, etc.) can also invite additional movements, and additional ways for the clients to tell the psyche’s stories. The sense of visibility in psychotherapy in general and in childcare in particular is crucial, and its control in front of the screen is obviously partial.

COVID-19 called for flexible changes and rapid adjustments on the part of the therapist in order to provide young children with continued support in spite of the instability of external realities. Our brief review of the literature highlights both the complexity of remote psychotherapy with children and its importance in view of the pandemic crisis. This pilot study was designed in response to the current crisis, in an attempt to make a contribution to the community, contribute to the understanding of the dynamics of remote psychotherapy, and fill the lacuna regarding the uniqueness of remote DMT with young children, specifically in group settings. The immediate objective of this project was to explore and identify the main intervention techniques which are available to therapeutic work in this situation.

## 3. Method

### 3.1. Participants

The current study involved eight girls and six boys aged six to seven. Participants were recruited through a message posted on media networks targeting parents of preschoolers. A third of the participating children had an early acquaintance with each other from their kindergarten setting. A snowball sample was also used, so parents and children who expressed a desire to participate in a Zoom DMT group referred the researchers to additional potential participants. All participants came from homes of a high–medium socioeconomic level, with no background of emotional/mental problems, as the DMT workshop was offered as a tool of prevention of future difficulties and as a means to process emotional contents which may arise at a time of crisis.

Parents were told that the DMT group is part of a study that examines the meaning of movement intervention in remote sessions aiming for emotional expression and support. It was explained that the purpose of the group is to allow space for personal and interpersonal expression in movement and verbal discourse in order to share emotional contents which the children are engaged with. One of the children stopped attending after the fourth session.

### 3.2. Tools

#### 3.2.1. Observation

Observations of two remote groups of DMT served as the central tool in the study. Both groups were identical in their structure (see details in Section 3.3) and were openly observed and registered by two DMT students. The observers were postgraduate DMT students during their second year of training in the M.A. program. During the first session, the participants were introduced to the observers so that they could feel comfortable with them, but the observers’ actual involvement in the meetings was minimal. The two observers took detailed notes and prepared transcripts of the sessions. The observers aimed for objective viewing as much as possible. One observer documented all the occurrences in the studied environment-objects, behavior patterns, conversations, and events in detail; the second observer paid special attention to the children’s bodies and their movement (changes in the use of space, movement intensity, diversity of movement) in order to provide an accurate description as far as possible [28]. This type of observation allows the collection of data during the meeting, reviewing the group processes and the various elements which come up both verbally and in movement. The observers also took personal notes after each session, recording their perception of it.

#### 3.2.2. DMT Student Diary

The workshop was led by DMT students during the second year of their postgraduate M.A. training. Two DMT students led each DMT group and documented their own responses to what had occurred regarding significant moments, choices, and decisions taken. Moreover, following each session, the session observers and the session leaders held a joint Zoom meeting in order to share insights that came up during the session. These were performed immediately after the session to allow associative thinking that contributes to the development of hypotheses, meanings, and themes that arose in the group. These discussions were also recorded in the diaries, served as data, and were later analyzed for this study.

### 3.3. Procedure

Parents who expressed interest in the group contacted the group leaders. After receiving an explanation about the nature and the aims of the project, a preliminary letter was sent out to the parents, detailing the dates and hours of the meetings, and requesting confirmations of consent. Parents were invited to call the group leaders prior to or between the sessions. Most parents expressed concern that the children would not persist in their participation (based on their previous experience with difficulties in remote educational settings following the COVID-19 restrictions). The DMT students who led the groups received supervision throughout the intervention from movement therapists with over twenty years of experience.

## 4. The Preparations and the Structure of the Meetings

As part of the data collection, for the research participants were divided into two groups and took part in six remote group DMT sessions (through Zoom). The small number of participants (seven children in each group) allowed for personal relations to develop between the children and the facilitators. Children who knew each other before the sessions were placed in the same group to allow them to feel safe and secure with familiar others. One group consisted of three children with early acquaintance, and in the other group two children had been previously acquainted with each other. The other children were distributed randomly. The groups were held during 2020, at a time when education frameworks were opened and closed intermittently. The data produced by both groups contributed to the triangulation and confirmation of the findings.

The sessions took place twice a week in the afternoon for a three-week period, with each session lasting 30–40 min depending on the attention span and the perceived ability of the participants. Before the meetings, the parents were asked to set up a computer with a camera and microphone for their children, and a private space where they could move freely without interruption. They were asked to prepare several permanent accessories for the sessions: a ball, a scarf/blanket, a pillow, drawing sheets, and paints. Prior to each session, a reminder message was sent to the parents.

## 5. The Group Intervention and Its Purpose

The meetings had a pre-set structure of opening, development, and closure, beyond which the therapists responded spontaneously and creatively with respect to children’s initiatives. The first meeting was introductory and consisted of explanations regarding the group framework and rules, the nature of the group (sharing and creating together), and familiarizing activities in order for the participants to become acquainted with one another and with the setting and its accessories. Each session began with a routine which included an opening song accompanied by movements, for the purpose of warm-up and a sense of group cohesion which would allow a shared experience to develop. At the core of the meeting, the therapists invited the participants to a movement experience in accordance with a central theme that emerged from the responses of the children.

The sessions dealt with topics that concerned the children in their daily lives through movement interventions such as movement reflection, spontaneous movement, movement with props, movement games, imagination, and verbal discourse. At the end of the session, the group leaders reflected on the main contents that emerged and invited the participants to share their feelings. Each session ended with joint group movement.

## 6. Data Collection

During the meetings two observers recorded the meetings, and later on, following each session, the observers and the group facilitators recorded their own feelings, thoughts, perceptions, and bodily sensations, and shared their personal impressions with one another. All these materials were used for the study.

## 7. Data Analysis

The specificity of remote group DMT with children was examined using interpretive phenomenological analysis (IPA) [29] of the session protocols (movement and words), and the personal diaries and notes written after each Zoom summary session. This method is unique in its focus on the subjective experience of the research subject along with its recognition of the role played by the investigator’s interpretation [30]. Data analysis was performed by experts in the field of DMT with children. During the first stage, all the diaries and meeting transcriptions were read and re-read, and notes were made regarding thoughts, observations, and reflections that occurred while reading. During the second stage, the transcripts and diaries were analyzed by identifying relevant topics and dividing topics into clusters, and a list of themes was compiled. Throughout the third stage, all the materials were again re-read to ensure that all topics were identified, which resulted in a complete list of relevant themes for each topic. In the fourth stage, the data and the topics were checked by an additional researcher experienced in use of the IPA method and by experts in DMT. At this stage, the participating students were invited to a focus group in which the themes uncovered were conveyed to them, and they were asked to share their feelings, emotions, and thoughts about them with the aim of gaining a more precise familiarity with the less conscious experiences [31,32].

## 8. Ethical Issues

The project was initially submitted to the in-house ethical committee on 10 February 2020, and was subsequently submitted to the faculty ethical committee of the University of Haifa and Seminar Hakibbutzim on 22 May 2022, both of which approved the proposal (approval no. 2968—the University of Haifa, and approval no. SUF2022_11—Kibbutzim College, Israel). The names used in this paper are pseudonyms to protect confidentiality.

## 9. Results

The study brought up three themes reflecting the remote intervention techniques and their impact in the enablement or hindrance of the creation of a safe space for personal expression. The first theme, “Play as a platform for transforming technical complexity into an expression of the inner world”, emphasizes the importance of providing symbolic meaning to the actions of the body, as a way to translate concrete behaviors into a symbolic game. The theme refers to the way in which limitations related to the use of technology and the distant encounter are expressed through the release of tension and emotional needs. The second theme, “Accessories and props as means of motivation for movement and imaginative play”, refers to the meanings generated through the use of projective objects and props from the children’s home as a framework for expression, sharing, and playfulness. The third theme, “Playfulness-inhibiting conditions in settings of remote therapy”, refers to situations and types of interventions that inhibit symbolic play in the group.

### 9.1. Play as a Platform for Transforming Technical Complexity into an Expression of the Inner World

One of the main themes which appeared in all the sessions was that by giving symbolic meaning to bodily actions, technical aspects related to the conditions of the remote encounter became central dynamic content in the sessions. This became evident through the participants’ preoccupation with revealing and hiding themselves to and from the group and the therapists throughout the sessions in several different ways: disappearing and returning to the screen (*n* = 12), hiding and revealing different body parts (*n* = 9), experimenting with turning the camera and microphone on and off (*n* = 10), discovery and concealment through sounds and voices (*n* = 8), changes in the computer display and shifting of it (*n* = 8), and the use of application filters (*n* = 14).

#### 9.1.1. Disappearing and Returning to the Screen

During all sessions, participants frequently left the camera while it continued to operate. The highest number of camera exits was observed during the first group meetings (*n* = 12). The reasons for these camera exits were going to the toilet, searching for an accessory the children wanted to show the others, calling a family member, or for no apparent reason.

For example, Andrew leaned down under the camera range so that he could not be seen. Following the therapist’s reference to his disappearance, Yael joined him and left the camera range, and following her, Michael hid behind his chair while turning it. This game was repeated throughout the sessions, especially at the beginning of the first sessions, when it was possible to notice the desire of the children for their names to be called out and their disappearance to be noticed. At times, the children peeked at the screen and went back into hiding, until their names were called out again. During the last group meetings, when the topic of the group ending came up, the pattern of disappearing and appearing returned more fully. For example, Debbie asked the therapists to hide and surprise the other children when they re-joined the group. As soon as Ben heard that more children were joining, he quickly jumped towards the screen and shouted “Boo!”. When he realized that Ellie had technical problems signing in and that she still could not see the group, he quickly hid again and, following his lead, John also hid.

#### 9.1.2. Exposure and Concealment of Body Parts

The opportunity to control and choose which body parts to hide or reveal allowed for a movement investigation to develop and for contents related to body image to arise. For example, in the first meeting, Ruth chose to get very close to the camera and show her face in zoom-in, thus hiding the rest of her body. Later in the session, during the time devoted to creative artwork, she turned the camera toward her desk so that only her page, her marker, and her drawing hand could be seen. In contrast, during the artmaking time in the third session, Neomi turned the camera very close to her body so that the screen would only show parts of her body: chest, neck, and half her face. An example of a group discussion on the participants’ body parts was seen in the fourth session: Neomi approached the camera with her eye; Layla followed her and said, “I prefer myself whole”. Afterwards, Debbie also approached closer to her camera, showed her mouth for a few seconds and then her nose and said, “I also prefer myself whole”.

#### 9.1.3. Turning the Camera On-off

The act of turning the camera off and on came up frequently. Certain situations were characterized by an experience of shyness and control. For example, during the first four sessions, Neomi turned her camera off while moving and turned it back on when the movement part ended. Other situations were mostly related to the experience of surprise and control, with transitions between a need for privacy and a sense of visibility. For example, in the last meeting, Gali turned off her camera in order to wear a dancer’s costume and put on make-up. She repeated this ritual and every time she turned her camera on, she appeared with a surprising new look.

The introduction of an accessory allowed for the expansion of the theme and the practice of discovery and concealment in a playful way, without turning off the camera.

Neomi placed an umbrella in front of her so that she was completely hidden by it, while occasionally peeking out in diverse ways. Michael and Guy chose to cover their faces completely using a scarf. Andy copied this routine and added, “we see and cannot be seen”. He also took a piece of paper and brought it close to his body, hiding with it various body parts. Following this theme, the therapists joined the children in the exploration and invited the group members to join their friends’ movements and explore different ways to hide body parts. The therapists proposed that the children should move the accessories either nearer to or away from camera and examine the effect on what can be seen and not seen on the screen, while at the same time helping the children understand what they see and do not see on their screens.

#### 9.1.4. Discovery and Concealment through Sounds and Voices

The participants chose to discover/hide by turning the speaker on and off. For example, Layla preferred to leave her microphone on at every opportunity, even when background noises in her home environment were heard at the meeting. In contrast, Kim and Sam preferred to turn on the microphone only at the moments when they spoke. Michael expressed frustration due to his failure to silence the microphone. He tensed his face muscles and held his gaze and body tightly. He raised his voice and asked: “Do you hear me? Do you still hear me?” He asked again in dissatisfaction with his unsuccessful attempts. Andrew spoke while his microphone was turned off, and he did not notice this until the therapist said that she could not hear him. Following this, he seemed embarrassed, turned his microphone on and asked, “now can you hear me?” over and over again. On one occasion, he approached the screen with a toy microphone, which emphasized the contrast between making his voice heard in the room and the fact that it was not heard in the group.

#### 9.1.5. Changes in the Computer Display and Shifting of It

Realistic events that evoked feelings which were difficult to self-contain (fear of change, separation, and rejection) were worked out through symbolic and imaginary play, using the camera and the screen. For example, in the second session, after Andrew’s camera moved quickly, Ben looked on in apparent confusion and apprehension, asking “why is Andrew’s house swaying?”, and then shook his whole body on purpose. His movement seemed playful as if he was looking for an unstable physical sensation of rickety and frightening ground. Michael, Andrew, Ben, and Yael moved the cameras with amusement and pleasure so that it would seem as if their houses were also shaking. This meeting took place at sundown when it was dark. Michael shared his fear of the darkness saying, “I’m a little scared”. Ben answered with a decisive tone, “There are no monsters yet, this is not a city of monsters”, trying to reassure both Michael and himself. The therapist encouraged this symbolic collaborative discourse as a way to process emotional experiences of uncertainty related to the outside real world and the inner fears.

#### 9.1.6. The Use of Application Filters

Digital means, such as Zoom app filters, allowed participants to change their appearance and dress up using imaginary bows, hats, and mustaches, and all the participants of the groups used these filters in all the sessions (*n* = 14). The use of filters evoked much laughter and a spirit of playfulness and stimulated the participants to move freely using their face gestures and upper body. For example, Neomi used a COVID-19 filter and said, “I took the child with the mask to a candy factory”.

### 9.2. Accessories and Props as Means of Motivation for Movement and Imaginative Play

Two types of accessories encouraged imaginative play: projective accessories such as a ball, scarf, and pillow, which the children were asked to bring to the meeting (*n* = 14), and personal accessories from the child’s room and surroundings (*n* = 13).

#### 9.2.1. Projective Accessories

The findings show that the objects that were prepared in advance were used both as a way of connection between the children and as a method for personal expression and the uniqueness of each one of them. Using these pre-prepared props (scarfs, balls) the facilitator encouraged expression of the inner world. For example, in the third session the group leaders encouraged the children to play as monsters and offered them the help of the scarf they had. Each child chose an image taken from their inner world. Ben said, “I am a green ghost”, as he covered himself with a green scarf and jumped between beds in his room. Michael was a “raven” who made “croaking” sounds while waving his hands and scarf. Neomi wrapped herself in a scarf like Michael and waved her scarf at the sides of her body, but her movements were gentler and smaller: “I am a bird,” she said, making bird sounds in a whisper. Later the kids wanted to become “Superman” and “Spider-Man” and show off their superpowers.

#### 9.2.2. Personal Accessories

The facilitators’ openness to the children’s use of personal belongings from their homes allowed for additional methods of communication and expression to develop within the group. For example, Andrew proudly showed up as “a floating robot”; Michael presented the “Book of the Dead-The Dinosaurs”; and Ben brought “a robot that can surf on ice, do stunts and flips in the air”. Demonstrating the abilities and superpowers of the characters they chose, the children’s bodies moved powerfully. Andrew and Ben jumped eagerly, their transitions in space were fast and surprising, and they moved quickly around their rooms while making enthusiastic noises. In other sessions, the participants dressed up as old people, carrying heavy baggage, bending over with their bodies, and offering each other imaginary bowls of soup.

### 9.3. Playfulness-Inhibiting Conditions in Settings of Remote Therapy

In both groups, most of the children expressed difficulty in playful and symbolic emotional expression in various situations throughout the sessions, especially during the first and last sessions of the intervention [10], in situations where feelings of insecurity arose [8], and when faced with structured guidance [12].

#### 9.3.1. Functioning of the Children as Affected by the Process of Acquaintance and Separation

The initial sessions were characterized by over-engagement with the app, and lack of attention, sharing, and playfulness. The first session was mainly characterized by the children’s interest in the functions of the Zoom program and getting to know the facilitators and the framework. As the framework of the sessions became more established and stable, the children felt increasingly confident, and more expressions with imaginative content were discovered. Alongside the difficulty to engage in the first sessions, expressions of ending in general and with regard to the Zoom session in particular also came up many times and affected the processes in the group. At the end of the meetings, resistance was expressed though anger, disappointment, and dissatisfaction. For example, Andrew asked, “why do we have to finish now when in kindergarten we have plenty of time?”. Ben expressed frustration that only a few of the accessories were used in the session and said, “so we just wasted the time when we took them”. Michael expressed anger towards the therapists, saying “Naughty you! You are Rude! We need to finish just when I wanted to play this song … Bye and never see you again!”.

The last session evoked regressive behaviors, as the children fiddled with personal objects while talking and refrained from looking straight at the screen, moving frantically. Michael said, “this is my worst day I have ever had in my life”. The children moved away and approached the screen, touched the body, or fell into fetal positions in the chair. The processing of sadness and difficulty in parting was also shown through the loss of pets that the children mentioned in the sessions. The children offered to show pictures of their pets that died and spoke of shared moments with them. Conversely, Ben especially looked for optimistic moments, found it difficult to experience sadness due to parting, and spoke about his plans after the sessions ended.

#### 9.3.2. Feelings of Insecurity

At moments when the external reality was uncertain and the distance from each other was especially present, no imaginary game took place. For example, Andrew fell from a chair in his house and was injured during one of the sessions. The children and the therapist were worried and expressed concern for his safety. In response, the children started playing hide and seek and the therapist reflected the emotional experience, “today it’s difficult to be together. Andrew fell in his house, and we could not keep him safe as we are far. We are both together and alone”. At these moments, the imaginary game stopped. In other cases, where there was a sense of concern for the safety of the children, for example when Ben jumped from bed to bed in his room while trying to fly, the therapists tried to gain control and warned the children of the danger. Here, too, the imaginary game stopped at that point.

Additionally, changes in the participants’ lives as a result of COVID-19 affected the collaboration in the sessions. For example, at a session which took place on the day the lockdown began, the children left kindergarten without prior preparation, and a third of them did not show up for the session. Neomi, who was usually excitedly eager to participate, agreed to join the session only after an encouraging phone call. Yael refused to come, and vehemently opposed the Zoom meetings. Michael wrapped himself in a scarf and made baby noises, put out his tongue, jumped out of turn and leaned on the chair with his body folded and far from the screen. Andrew put various small objects into his mouth; Ben sat in a darkened room; Neomi kept quiet through most of the session and seemed to be not in the mood for anything. Sam was over-aroused and had difficulty regulating himself.

After entering another lockdown, Yael’s mother wrote to the therapists: “Yael doesn’t want to participate. She says it’s too short and ends quickly, and then she is sad. She is now also always sad when she sees her grandparents and then they leave, or when something is over”. In this session, Michael brought up the death of his cat without prior connection, “you know my cat is dead … she is no longer in my house …”. He later showed a book he had at home about extinct dinosaurs and explained that they were also dead. Thus, alongside the avoidance that the changes brought to the group, attending meetings allowed for the processing of difficult content and a space for sharing fears and feelings of helplessness, loss, and separation.

#### 9.3.3. Structured Guidance

It was noticed that when the therapists’ instructions were more specific, i.e., “shake the scarf and stop,” or “bring the ball closer to the screen,” the children mostly cooperated, following which the group synchronized, but there were no expressions of imaginary symbolic play.

## 10. Discussion

In DMT, body and movement are used as a way of communicating experiences that have not yet undergone representation. Associative movement invites an encounter with concealed areas of the psyche [33,34]. The findings of the study show that imaginary and emotional content is also expressed in the body in a group setting that takes place in a remote setting visible on the computer screen. The study indicates that attributing meaning to the children’s actions in the context of the new format of the meetings (screen boundaries, speaker and voice control, the mobility of the computer and the various filters) and translating their behaviors into emotional needs which are playfully expressed led to an expansion of the children’s emotional expression during the meeting. Furthermore, the type of objects and accessories spontaneously used by the children in their home setting and those pre-prepared by the facilitator (identical projectable objects) allowed for playfulness, imaginative work, and verbal discourse. At the same time, however, these interventions were not as effective before the establishment of an acquaintance with the facilitator, or at times of stress and in the face of closed structured instructions. The following discussion expands on the findings and formulates some practical conclusions for psychotherapeutic interventions when working with children in a remote setting at times of crisis.

## 11. Discovery and Concealment of the Body

The findings of the study indicate the participants’ extensive involvement in the issues of discovery and concealment/presence and absence. The content appeared through actions whose nature was regressive somatic-sensory, which explore the contrast between the “presence” and the “absence” of the body in the encounter with an observing other. Through the disappearances from the screen and the return to it, as well as the hide-and-seek games, the children practiced the experience of presence and disappearances in a real and concrete way. The game allowed the children to express still unverbalized experiences through their body movement and actions. The presence of the therapists through their gaze, reflection, and resonance gave this experience validity, meaning, and qualities of communication. As the meetings took place from afar through Zoom, we assume that the children’s acts of disappearance and discovery were triggered by their awareness of the physical distance that the lockdown brought with it. It seemed difficult for the children to symbolically hold the therapist’s presence in mind as a caring, interested, and attentive individual who is there for them. In situations where, in Winnicott’s (1963) [35] words, the child is unable to use the object when they are not physically next to it, and to feel protected and safe when not all their senses can recognize it, regressive behaviors may appear. In the sessions presented, we suggest that the children recreated initial regressive experiences, expressed in a somatic-sensory play of presence and absence. Themes of discovery and concealment through hide-and-seek games often appear in psychotherapy as a way of processing developmental processes of separation. This resonates with Freud’s (1920) “Fort-Da” (here/gone) game, where the infant masters separation by converting the passive experience into an active one [36]. It is also similar to the “peek-a-boo” game, where the child covers his/her face and “disappears” and then returns with increasing and decreasing stress levels. This allows the baby to practice gaining control over the mother’s disappearance and return as part of achieving a sense of object permanence [37].

It can be assumed that the physical distance from the therapist and the therapy room due to the pandemic brought up contents related to instability and insecurity as result of the circumstances, which made for some uncertainty as to the actual existence of the other. Through the hide-and-seek games, the children were able to re-process fears and needs related to internalizing the other’s presence, even when the other was not physically close.

In the present study, the theme of “discovery and concealment” may also be interpreted as avoidance and resistance, but in fact, the therapists highlighted the playful and creative qualities that the children expressed and encouraged the development of this content. References to acts of discovery and concealment through witnessing, observing, and echoing without providing interpretation presented the possibility for participants to continue to explore, to be intrigued, and express their emotions more consciously, as their motor expression also developed and became more complex. The legitimacy given to hiding and discovery through movement transformed the meaning of these behaviors from expressions of a defense mechanism to a method of communication, creativity, and play, which, in turn, led to the attainment of a sense of belonging to the group. Furthermore, through acts of discovery and concealment the participants controlled the level of intimacy and the degree of closeness and exposure and played with them. The sense of visibility in psychotherapy in general, and in children’s psychotherapy in particular, is essential, and its attainment in front of the screen is necessarily more complex and partial. In remote psychotherapy, clients are preoccupied with whether they can be seen, found, heard, felt, and protected—even more intensely than in the secure protective setting of the therapy room [24,26]. As mentioned, throughout the sessions, the therapists and the group’s attitude towards the acts of disappearance performed by the children was of great significance. The children seemed to have re-experienced this game when they needed to be looked at, noticed, heard, and reinforced. This was especially evident at the introductory stages and when issues of parting came up.

## 12. The Use of Accessories and Props as Projective Objects Which Regulate Emotion

Remote psychotherapy for children is a challenge due to the great mobility that characterizes the developmental stage of children, their limited span of attention, and the children’s need for non-verbal communication [15]. Accessories serve as mediators and bring children closer to a shared group experience, while still serving as means of expression for the children’s subjective experience [6]. Similarly, in the present study the accessories helped participants to be involved and share emotions. The use of personal or technological accessories supported group cohesion and a sense of belonging and served both as a container which holds and gathers the group together and as a means of expressing emotional content from the inner world of the children. In DMT, the use of accessories and props such as scarves and balls is very common, as these can offer sensory stimulation and be used to make connections without making physical contact [6]. The present study emphasizes the importance of using projective accessories that encourage movement for a sense of closeness even in remote, online therapy. The participants’ use of accessories which support movement (ball, fabrics, ropes), and the frequent use of the Zoom filters on the children’s own initiative, created many situations in which all participants collectively engaged in the same accessories. The props allowed participants to externalize their emotions through movement and thus process experiences in a less threatening way. When participants were given the opportunity to bring an object of their choice, they often chose a soft object that accompanied them throughout the session and helped reduce stress. It is possible that the use of an object compensated for the distance from the group members and the therapist, in the same way that the transitional object helps the child in separation while moving away from the mother [38].

The use of props also supported the expression of painful emotions, such as sadness, confusion, and helplessness. Many children chose to play act as elderly characters who are forced to carry heavy weights on their backs. It is thus safe to assume that the shared creative experience allowed the children to express their innermost fears which the global pandemic brought to their daily lives: the confrontation with uncertainty and the fear for relatives. The children’s act of making and offering imaginary bowls of soup to each other highlighted the power inherent in working through movement and accessories and expressed the children’s need for nourishment by and concern for their loved ones.

Through the therapeutic work with accessories, communication took place between the group members, which accorded with previous findings in clinical literature [4]. Using the props, the participants suggested movement activities to the group and invited the group members to join their imaginary world, as a way to further explore, process, and feel less alone. Taking on such an informal facilitation role by group participants may contribute to their growth process [6].

## 13. Processing of Psychological Content Related to COVID-19 through Remote DMT

The consequences of COVID-19, such as the closures of educational institutions which prevented children from meeting their peers and the possibility of Zoom therapy, affected the emotional content with which children were engaged in the sessions. Topics such as death, loss, and separation were expressed through physical absence, through various actions in the encounter, and in the verbal content expressed during the session. Participants were supported by the group and used it to process feelings of fear, anger, and sadness, to experience ego forces, and strengthen the sense of agency. Response to separation and termination appeared in spontaneous movements of venting and physical in-drawing, indicative of feelings of insecurity [39], e.g., making baby noises, wrapping oneself in a scarf, putting objects in the mouth, motions of frenzy and arousal, along with lack of movement and reduced liveliness.

Strong and fast movements appeared in the face of threatening situations, for example, when it seemed to Ben that the house was shaking and unstable, he converted the fear and confusion into movement, and embodied the same frightening tremor. Fear thus became a comic game joined by all team members. This reinforces the understanding in the literature that children use frightening or dangerous metaphors, such as monsters and dragons, and the pretense of magical ability to gain control of the fear of the unexpected and to interpret and process experiences [40]. This research shows that through the moving body, processing emotional experiences is also possible through online, remote therapy, despite the limitation of screen boundaries and the distance among participants and between them and the facilitators.

## 14. Practical Implications

It is essential to arrange for an inclusive, containing environment in remote group DMT for children, an environment where trust and confidence may be built regardless of the physical distance of the therapists and the absence of clinical frameworks. This type of environment will allow for transitional experiences between the children’s inner psychic reality and the outer reality. These experiences are important for processing emotions indirectly through the assembly of stories, images, and metaphors, which also considerably affect movement expression, movement diversity, and quality of movement. It is our understanding that in this type of therapy and circumstances the therapists should give special attention to:

1. Interweaving educational observation and symbolic play—endorsing the role of the therapist as a supporter of conscious translation to unconscious movement.
2. The functional use of media and technology and its projective use to allow the children to lead and show the therapist the endless possibilities of telling the psyche’s tale.
3. Using the entire space of the room for movement work—children need space beyond the boundaries of the computer.
4. The projective use of accessories in movement, as a way to support the expansion of movement and get in touch with inner psychic materials.
5. Listening both to the verbal and the non-verbal content that arises in remote DMT sessions enables contact with the children’s’ emotional experiences, especially those related to coping with stress and changes that the COVID-19 outbreak brought with it.

Notably, the study found that in situations of stress, the therapist’s ability to hold the projective and potential space for the children is limited, and that keeping the sense of safety is challenged. Given the daily pressure of such situations, which also effects the stability of home settings, remote therapy may also be less effective. It is precisely in these situations that face-to-face interventions are critical, and remote online work is at best a default intervention in the absence of other options.

## 15. Limitations and Future Studies

The study was conducted with a small sample of participants with similar characteristics, and the qualitative data were based on the therapists’ experience and their impression of the process in the sessions. Additionally, the scope of the meetings was limited. This should be further explored, and the sample size and research tools should be expanded. The participants’ point of view and their perceptions of the experience should also be explored and evaluated. Furthermore, the study addresses both the proactive and preventive aspects which can be worked with in remote therapy as well as the importance of studying a population without particular emotional difficulties, as a way of recognizing the uniqueness of the remote DMT sessions with young children. Further studies may relate to other populations with identified difficulties and other diagnoses. Moreover, as we reasoned that it was beneficial for M.A students to have experience in delivering therapy remotely during their studies, second-year DMT students delivered the sessions and observations. We predicted that competency and efficacy in this area could have important professional and public health implications and that acquiring enhanced clinical skills could later be potentially beneficial for delivering remote services. That said, further studies should involve more experienced and certified therapists who facilitate and study the remote DMT group intervention. Finally, it is important to examine the impact of remote therapeutic sessions versus the impact of face-to-face encounters by examining the clients’ feelings and the extent of observable changes before and after the intervention.
